# Cardiovascular factors moderate the association of infection burden with cognitive function in young to middle-aged U.S. adults

**DOI:** 10.1371/journal.pone.0218476

**Published:** 2019-06-13

**Authors:** Dawson W. Hedges, Andrew N. Berrett, Lance D. Erickson, Bruce L. Brown, Evan L. Thacker, Shawn D. Gale

**Affiliations:** 1 Department of Psychology, Brigham Young University, Provo, Utah, United States of America; 2 The Neuroscience Center, Brigham Young University, Provo, Utah, United States of America; 3 Department of Sociology, Brigham Young University, Provo, Utah, United States of America; 4 Department of Public Health, Brigham Young University, Provo, Utah, United States of America; Universidade de Sao Paulo, BRAZIL

## Abstract

**Background:**

Infectious diseases might affect cognitive aging and dementia risk, possibly via neuroinflammation. Similarly, risk factors for cardiovascular and cerebrovascular diseases are associated with cognitive function and dementia. We hypothesized that cardiovascular risk factors moderate the association of exposure to infectious diseases with cognitive function.

**Methods:**

We studied 5662 participants aged 20 to 59 years from the third National Health and Nutrition Examination Survey (1988–1994) in the United States. We used linear regression to investigate whether the Framingham general cardiovascular risk index moderated the association of infection burden based on exposure to eight different infectious diseases with cognitive functioning as measured by the Symbol Digit Substitution, Serial Digit Learning, and Reaction Time tests.

**Results:**

The multiplicative interaction between the infection-burden index and the cardiovascular-risk index was associated with performance on the Symbol Digit Substitution (B = .019 [95% CI: .008, .031], *p* < .001) but not on the Serial Digit Learning (B = .034 [95% CI: -.025, .094]) or for Reaction Time (B = -.030 [95% CI: -.848, .787]). Participants with a lower cardiovascular risk appeared to be more resilient against the potential adverse effects of higher infection burden on the Symbol Digit Substitution task.

**Conclusions:**

Participants at zero risk for a cardiovascular event in the next 10 years had no differences in processing speed with increasing exposure to infectious disease, whereas participants with higher risk for a cardiovascular event had worse processing speed with increased exposure to infectious disease.

## Introduction

Numerous factors influence cognitive function. Although increasing age is a strong risk factor for Alzheimer’s disease, sex [1] and genetic factors such as APOE ε4 status are also associated with Alzheimer’s disease [2]. Depression in late life, diabetes, hearing loss, mid-life hypertension, low educational attainment, mid-life obesity, low levels of physical activity, smoking, and social isolation also appear to be risk factors for dementia [3, 4]. Among the risks associated with cognitive deficits and dementia are cardiovascular factors [5, 6] and exposure to infectious disease [5, 7–9].

Certain viral, bacterial, and parasitic exposures appear to be associated with deficits in cognitive function. Findings suggest that seropositivity for *Toxoplasma gondii* (*T*. *gondii*) or *Toxocara canis* and *cati* might adversely affect processing speed [10, 11]. Seropositivity for the bacterium *Helicobacter pylori* (*H*. *pylori*) [12] is also adversely associated with cognitive function. Moreover, exposure to certain infectious diseases has been associated with Alzheimer’s disease. In this regard, a meta-analysis found increased odds of developing Alzheimer’s disease based on the presence of herpes virus type 1 and Epstein-Barr virus DNA in the brain, a risk further increased in APOE ε4 carriers [8]. One possible link between infection and cognition is the local or systemic inflammation that infectious diseases initiate [13].

Factors that affect cardiovascular health also pose a risk to cognitive function. Knopman et al. [14] found that hypertension and diabetes were associated with cognitive decline over a six-year period in adults aged 47 to 57 years. A more recent study found that the aggregate effects of cardiovascular risk factors present in children and adolescents were associated with lower cognitive function in adulthood [15]. Similarly, a seven-factor cardiovascular health score including smoking, body mass index, physical activity, diet quality, blood pressure, blood cholesterol, and blood glucose has been shown in multiple studies to be associated with cognitive aging [16, 17]. Obesity also appears to be associated with Alzheimer’s disease [6]. Finally, a systematic review found that various cardiovascular disease prediction models, including Framingham risk models, are associated with changes in cognition over time [5].

At least some cardiovascular-risk factors such as diabetes and hypertension might compromise the blood-brain barrier and thus permit entry of neurotoxins [18] and possibly infectious pathogens into the brain [19]. Moreover, compromised function of the blood-brain barrier has been associated with dementia [20]. In addition, certain infectious diseases themselves might be associated with early cardiovascular disease [21].

Because risk factors for cardiovascular disease might alter the function of the blood-brain barrier and because compromised blood-brain barrier function might lead to an influx of toxins including infectious diseases into the brain, we hypothesized *a priori* that cardiovascular risk moderates the association of exposure to infectious diseases with cognitive function in young to middle-aged adults. To test our hypothesis, we sought to determine whether a multiplicative interaction between cardiovascular risk and infection burden predicts cognitive function.

## Materials and methods

### Study sample

Conducted by the Centers for Disease Control and Prevention (CDC) in the US from 1988 to 1994, the third National Health and Nutrition Examination Survey (NHANES III) is designed to represent the non-institutionalized US population by using statistical weighting and stratified sampling techniques. Overall and component-specific response rates for the NHANES III are available online at https://wwwn.cdc.gov/nchs/data/nhanes3/ResponseRates/nh3_rr.pdf. Of the almost 40,000 participants originally recruited, NHANES III researchers interviewed 86% and examined 78%. Because the NHANES III assessed only a subsample of the participants aged 20 to 59 years for cognitive function, our total sample was the 5662 participants between the ages of 20 to 59 years whom the NHANES randomly selected for the cognitive assessments. Data in this study were collected by the U.S. government (National Center for Health Statistics, which is part of the CDC) in compliance with all federal laws concerning ethical guidelines including obtaining informed consent. Data are anonymized and freely available online. Although more recent datasets are available from the CDC, we used the NHANES III dataset because it was the most recent NHANES that contained the necessary immunological and cognitive variables necessary to test our hypothesis.

### Infection-burden index

We obtained data for infectious pathogens in both phases (1988–1991 and 1992–1994) of the NHANES III datasets, which included *Toxocara* species, *Toxoplasma gondii*, cytomegalovirus (CMV), herpes simplex virus 1, herpes simplex virus 2, hepatitis A, hepatitis B, and hepatitis C. CDC laboratory technicians originally obtained and tested the blood and serum samples from all consenting participants. Because the hepatitis A vaccine was not available in the US until after data collection in the NHANES III [22], seropositivity for antibodies to hepatitis A in this sample represents past exposure to hepatitis A and not vaccination. A more detailed description of the laboratory methodologies used to identify infection is available in the NHANES III laboratory manual [23]. Briefly, for *Toxocara*, *T*. *gondii*, CMV, hepatitis A, and hepatitis C, CDC laboratory technicians determined seropositivity by an enzyme immunoassay in which they combined the samples with antigen, labeled anti-human IgG, and a substrate to initiate a color reaction proportional to the concentration of pathogen antibodies in the blood sample. The CDC technicians detected antibodies specific for herpes simplex 1 and 2 via solid-phase enzymatic immunodot assay. In this test, the CDC technicians added purified gG-1 or gG-2 glycoproteins to the center of a nitrocellulose disk and incubated test serum with the disk. Extensive washing reduced cross-reactivity with other non-specific antibodies. The addition of anti-human IgG and an enzyme substrate initiates a color reaction if antibodies are present in the sample. Finally, the CDC technicians determined the presence of hepatitis B antigen by sandwich radioimmunoassay. In this test, two anti-hepatitis B antibodies, one of which conjugated to ^125^I, sandwiches any hepatitis B antigen present in the test sample. A gamma counter can then detect this antibody-antigen-antibody-^125^I complex.

To generate a measure of overall infection burden, we summed the total number of pathogens for which each participant was seropositive, consistent with the method Miller et al. [24] used. With this method, a subject seropositive for hepatitis B and *Toxocara* but no other pathogens would receive an infection-burden score of two. Other methods exist that can estimate overall infection-burden index [25, 26], but the referenced methods base the infection-burden index on the sum of regression coefficients obtained from analyses of each pathogen and the outcome variables of interest, such as cognition. In our case, however, we sought to determine whether the cardiovascular-risk index moderated the association between the infection-burden index and cognitive outcome. As such, we chose to use a method of estimating the infection burden that is independent of the outcome variables. However, we performed all analyses using the count method as well as the method described by Elkind et al. [27]. Both methods produced nearly identical results.

### Cardiovascular-risk index

Multiple methods currently exist for constructing cardiovascular-risk indices designed to predict specific cardiovascular outcomes such as stroke or myocardial infarction [5]. Because our objective was to explore interactions between infection burden and general cardiovascular health, we used the Framingham general cardiovascular risk profile developed by D’Agostino et al. [28] for the purpose of predicting any cardiovascular-disease event (composite of any coronary heart disease, any cerebrovascular event, peripheral vascular disease, and heart failure) as opposed to a singular cardiovascular outcome. Based on a series of Cox proportional-hazards regressions, the Framingham general cardiovascular risk index predicts a 10-year estimate of risk for any cardiovascular-disease event from multiple risk factors including age, total cholesterol, high-density lipoprotein, systolic blood pressure (separated into two factors based on whether an individual took antihypertensive medication or not), cigarette-smoking status, and diabetes. From these estimations, D’Agostino et al. [28] constructed sex-specific reference tables used to calculate a general 10-year cardiovascular risk percentage. Using these reference tables [28], we calculated the 10-year cardiovascular risk percentages for all men and women in our study. We centered the cardiovascular-risk index for the statistical analyses to avoid problems of collinearity when modelling its interaction with the infection-burden index.

### Cognitive function

NHANES technicians administered the Symbol Digit Substitution test, the Serial Digit Learning test, and a simple Reaction Time test to a subsample of NHANES participants to assess cognitive functioning. A detailed description of these measures is available elsewhere [29]; briefly, however, the Symbol Digit Substitution test is a computerized task that requires participants to use a key at the top of the screen to match a series of nine digits to their corresponding symbols in a two-minute period for a total of four trials, recording and summing the total latency (in seconds) between each digit-symbol pairing for each trial. The final score is the average of the two lowest total latencies from the four trials. Therefore, a lower score on the Symbol Digit Substitution test indicates better performance. In general, the Symbol Digit Substitution test is a measure of processing speed. The Serial Digit Learning test is a measure of short-term memory, earning, and attention in that it requires participants to memorize and recall a string of eight random digits. The participants have nine attempts (or trials) to recall the series of digits. For each trial, participants receive two points if they recall less than two-thirds of the digits, one point if they recall more than two-thirds of the digits, and zero points if they correctly recall all the digits. Participants who correctly recall the digits for two consecutive trials do not proceed onto the next trial. A participant’s score on the Serial Digit Learning test is the total number of points received across all completed trials. Therefore, as with Symbol Digit Substitution, a lower score indicates better performance. For Reaction Time, participants pressed a button on a designated response device each time a small, square-shaped stimulus appeared on the computer screen in front of them. After excluding the first ten trials and any trial with values greater than 750 milliseconds or less than 50 milliseconds, the mean amount of time between stimulus onset and button press (in milliseconds) over 50 trials was the overall Reaction Time score. As with Symbol Digit Substitution and Serial Digit Learning, a lower Reaction Time score indicates better performance. We used raw scores from all three cognitive measures. There are no standardized scores available because cognitive function in the NHANES dataset is associated with the same sociodemographic variables that we used as control variables in our models [29].

### Control variables

We included race-ethnicity, sex, age, poverty-to-income ratio (PIR), and educational attainment in all analyses as control variables to account for potential confounding. Race-ethnicity categories included non-Hispanic White, non-Hispanic Black, Mexican American, and Other. The PIR is a continuous variable calculated by dividing a participant’s income by the federal poverty level at the time of the survey. For example, a PIR value of three indicates that a participant’s income is three times the federal poverty level. The NHANES measured age in years and educational attainment as the total number of years of formal education.

### Statistical analyses

Prior to analyses, we treated missing data with multiple imputation using chained equations. Multiple imputation requires the Missing at Random assumption for missing data, which is less strict and more realistic for most survey data than are traditional methods of addressing missing data, such as complete case analysis/listwise deletion and mean substitution. The chained-equations approach allows for the prediction equation for the missing data in each variable to be consistent with its distribution (e.g., Ordinary Least Squares regression for continuous variables, logistic regression for dichotomous variables). Current recommendations suggest that the number of imputations should be at least the same as the percentage of participants with missing data [30]. Because approximately 50 percent of participants were missing at least one value, we used 50 imputed datasets to meet the recommendations for number of imputations. Herpes 1 and herpes 2 were always missing simultaneously, and they were the variables with the most missing data with 26 percent of their values being imputed. The next variable with the most missing was Serial Digit Learning with 12 percent imputed. The mean percent imputed for all variables included in the study was 8.35 with a standard deviation of 7.81. Datasets were separated by 200 iterations based on graphical diagnostics that indicated the imputation model converged well before that [31]. Because the infection-burden index and cardiovascular risk were aggregate measures in our analyses, we imputed the individual items and computed the aggregate measures after imputation.

We conducted statistical analyses using Stata 15.0 [32] and used the built-in *svy* prefix to account for the NHANES weighting, strata, and cluster variables. We computed means and standard errors for all continuous variables and proportions and standard errors for categorical variables to characterize the study sample. We used Ordinary Least Squares regression to test whether the interaction between the infection-burden and cardiovascular-risk indices was associated with performance on the Symbol Digit Substitution, Serial Digit Learning, and Reaction Time tests and included all control variables in each analysis. We also checked that the statistical models were consistent with the assumptions of Ordinary Least Squares regression. The assumptions held in most cases. There was slight evidence of heteroskedasticity, although the inferences from the model were robust to this (i.e., results from the naïve model and model accounting for heteroskedasticity were substantively identical) and report the naïve models. We centered the infection-burden and cardiovascular risk indices for the statistical analyses because the introduction of their interaction resulted in variance inflation factor scores above 5. Because of the potential for alpha inflation and risk of a type-1 error due to multiple comparisons, we subjected our findings to the Benjamini-Hochberg correction with a false-discovery rate set at 10 percent [33].

Because the cardiovascular-risk index reference tables [28] provided were sex-specific, we tested for interactions effects between the infection-burden and cardiovascular-risk indices for men and women separately as a post-hoc exploratory analysis to determine if any effects were sex-specific. In post-hoc exploratory analyses, we tested whether cardiovascular risk moderated the association between an infection-burden index based on only IgG titers to the available viruses and cognitive function as well as whether cardiovascular risk moderated the association between an infection-burden index based on only IgG titers to the available parasites and cognitive function. We did not apply the Benjamini-Hochberg correction to the post-hoc analyses.

## Results

The unweighted sample size was 5662. In regard to demographic characteristics, 74% of the study sample were non-Hispanic White, and 49% were female. On average, participants had obtained at least a high-school education and were earning incomes three times greater than the US federal poverty level. The most common infection was herpes simplex type 1, with 67% of the study sample testing as seropositive. Hepatitis C was the least common infection with only 3% of participants being seropositive (Table 1).

**Table 1:** Unweighted summary statistics of study sample.

	Full Sample		Men		Women	
	Mean or Proportion	SE	Mean or Proportion	SE	Mean or Proportion	SE
Cognitive Function						
Symbol Digit Substitution	2.71	.03	2.77	.03	2.65	.03
Serial Digit Learning	4.67	.16	4.61	.18	4.73	.18
Reaction Time	234.23	1.37	226.34	1.72	241.82	1.88
Infection Burden	2.18	.04	2.10	.05	2.25	.05
Infectious Diseases						
*Toxocara*	.14	.01	.16	.01	.12	.01
*Toxoplasma gondii*	.20	.01	.21	.01	.19	.01
Cytomegalovirus	.57	.01	.52	.02	.63	.02
Herpes Simplex Virus 1	.67	.01	.65	.02	.69	.02
Herpes Simplex Virus 2	.24	.01	.19	.01	.28	.01
Hepatitis A	.28	.02	.29	.02	.28	.02
Hepatitis B	.05	.01	.06	.01	.04	.01
Hepatitis C	.03	.00	.04	.01	.01	.00
Percent of Cardiovascular Risk	2.51	.06	3.01	.08	2.02	.06
Age	37.10	.23	37.02	.29	37.18	.27
Female	.51	.01	-	-	-	-
Race-ethnicity						
Non-Hispanic White	.74	.02	.75	.02	.74	.02
Non-Hispanic Black	.12	.01	.11	.01	.13	.01
Mexican American	.06	.01	.06	.01	.05	.01
Other	.08	.01	.08	.01	.08	.01
Education (years)	12.67	.09	12.69	.10	12.65	.09
Poverty-to-income Ratio	3.07	.08	3.12	.08	3.01	.10

N = 5662

Abbreviations: SE = Standard Error, SDS = Symbol Digit Substitution, SDL = Serial Digit Learning

We found that the interaction between the infection-burden index and the cardiovascular-risk index was associated with performance on the Symbol Digit Substitution test (B = .019 [95% CI: .008, .031], *p* < .001). This remained significant after adjustment for false discovery with the Benjamini-Hochberg test [33]. Participants who had a lower cardiovascular risk appeared to be most resilient against the potential adverse effects of higher infection burden on Symbol Digit Substitution performance. For example, among participants who scored zero on cardiovascular risk, there was no association of infection burden with Symbol Digit Substitution test (B = -.002 [95% CI: -.037, .033], *p* = 0.910). However, people with 2.5 percent cardiovascular risk (the mean value of cardiovascular risk) were predicted to have Symbol Digit Substitution score 0.047 points higher (95% CI: .021, .072; *p* = .001) per 1-point increment in infection-burden index. Similarly, people with 5 percent cardiovascular risk were predicted to have Symbol Digit Substitution score .095 points higher (95% CI: .055, .136; *p* < .001) per 1-point increment in infection-burden index. People with 10 percent cardiovascular risk were predicted to have Symbol Digit Substitution score .193 points higher (95% CI: .102, .283; *p* < .001) per 1-point increment in infection-burden index. We did not find significant interactions between the infection-burden index and the cardiovascular-risk index on the Serial Digit Learning test (B = .034 [95% CI: -.025, .094]) or for Reaction Time (B = -.030 [95% CI: -.848, .787]) (Table 2).

**Table 2:** Interaction effects of an infection-burden index and cardiovascular-risk index and association with cognitive function in U.S. adults aged 20 to 59 years.

	Symbol Digit Substitution		Serial Digit Learning		Reaction Time	
	B	95% CI	B	95% CI	B	95% CI
Infection Burden Index^a^	.047***	[.021,.072]	.196**	[.064,.327]	.512	[-1.254,2.279]
Cardiovascular Risk Index^a^	-.000	[-.019,.019]	.029	[-.061,.118]	-.563	[-1.485,.358]
Interaction	.019***	[.008,.031]	.034	[-.025,.094]	-.030	[-.848,.787]
Age (years)	.030***	[.027, .033]	.089***	[.072, .105]	.461***	[.274, .648]
Female	-.151***	[-.212, -.091]	.039	[-.277, .355]	14.235***	[9.627, 18.842]
Race-ethnicity						
Non-Hispanic White (ref)	---	---	---	---	---	---
Non-Hispanic Black	.360***	[.293, .427]	1.666***	[1.252, 2.081]	12.675***	[7.333, 18.016]
Mexican American	.261***	[.156, .366]	2.207***	[1.645, 2.769]	6.776*	[.340, 13.212]
Other	.321***	[.156, .485]	2.148***	[1.209, 3.087]	4.011	[-6.139, 14.161]
Education (years)	-.119***	[-.134, -.104]	-.519***	[-.585, -.453]	-2.719***	[-3.748, -1.690]
Poverty-to-income Ratio	-.037***	[-.051, -.023]	-.262***	[-.363, -.162]	-2.350***	[-3.305, -1.395]
Constant	3.212***	[2.995, 3.429]	8.231 ***	[7.129, 9.333]	249.340***	[235.119, 263.561]

N = 5662.

^a^ The indexes were centered to reduce collinearity in the presence of their interaction.

Abbreviations: CI = Confidence Interval, ref = Reference Category

*p* < .05 *

*p* < .01 **

*p* < .001 ***

The same analyses conducted separately for women and men showed a potential sex-specific effect in that the interaction effect between the infection burden and cardiovascular-risk indices for Symbol Digit Substitution was present in women (B = .032 [95% CI: .015, .048], *p* < .001) (Table 3) but not in men (B = .012 [95% CI: -.004, .028]) (Table 4). The interaction in women remained significant after adjustment for false discovery due to multiple comparisons using the Benjamini-Hochberg test [33]. However, in a post-hoc exploratory analysis, there was not a statistically significant three-way interaction effect between the infection burden index, cardiovascular-risk index, and sex (B = .02 [95% CI: -.00, .04]).

**Table 3:** Interaction effects between an infection-burden index and cardiovascular-risk index and associations with cognitive function in female U.S. adults aged 20 to 59 years.

	Symbol Digit Substitution		Serial Digit Learning		Reaction Time	
	B	95% CI	B	95% CI	B	95% CI
Infection Burden Index^a^	.057**	[.022,.092]	.265**	[.077,.454]	.244	[-2.255,2.743]
Cardiovascular Risk Index^a^	-.000	[-.024,.024]	.084	[-.095,.263]	-.037	[-1.762,1.687]
Interaction	.032***	[.015,.048]	.038	[-.056,.133]	-.423	[-1.375,.529]
Age (years)	.031***	[.026,.035]	.090***	[.064,.116]	.409**	[.131,.687]
Race-ethnicity						
Non-Hispanic White (ref)	---	---	---	---	---	---
Non-Hispanic Black	.370***	[.272,.467]	1.584***	[1.056,2.111]	15.474***	[9.218,21.731]
Mexican American	.421***	[.261,.581]	2.509***	[1.819,3.199]	13.231**	[4.333,22.129]
Other	.389**	[.160,.617]	2.513***	[1.134,3.891]	3.357	[-10.557,17.271]
Education (years)	-.108***	[-.131,-.086]	-.485***	[-.573,-.396]	-3.016***	[-4.639,-1.394]
Poverty-to-income Ratio	-.053***	[-.077,-.029]	-.292***	[-.429,-.156]	-2.051**	[-3.502,-.599]
Constant	2.918***	[2.591,3.245]	7.863***	[6.450,9.275]	268.167***	[245.929,290.404]

N = 3068.

^a^ The indexes were mean centered to reduce collinearity in the presence of their interaction.

Abbreviations: CI = Confidence Interval, ref = Reference Category

*p* < .05 *

*p* < .01 **

*p* < .001 ***

**Table 4:** Interaction effects between an infection-burden index and cardiovascular-risk index and associations with cognitive function in male U.S. adults aged 20 to 59 years.

	Symbol Digit Substitution		Serial Digit Learning		Reaction Time	
	B	95% CI	B	95% CI	B	95% CI
Infection Burden Index^a^	.046**	[.013,.080]	.124	[-.070,.319]	.449	[-1.936,2.835]
Cardiovascular Risk Index^a^	-.005	[-.030,.019]	-.023	[-.118,.072]	-.912	[-2.178,.354]
Interaction	.012	[-.004,.028]	.045	[-.022,.112]	.294	[-.900,1.488]
Age (years)	.029***	[.025,.034]	.087***	[.063,.110]	.494***	[.233,.755]
Race-ethnicity						
Non-Hispanic White (ref)	---	---	---	---	---	---
Non-Hispanic Black	.337***	[.238,.436]	1.712***	[1.102,2.321]	9.550*	[1.919,17.181]
Mexican American	.112	[-.001,.225]	1.924***	[1.185,2.662]	.977	[-7.268,9.222]
Other	.250*	[.018,.482]	1.762**	[.606,2.918]	4.950	[-9.814,19.714]
Education (years)	-.130***	[-.151,-.109]	-.554***	[-.649,-.459]	-2.499***	[-3.763,-1.236]
Poverty-to-income Ratio	-.021	[-.045,.002]	-.228*	[-.407,-.049]	-2.499***	[-3.857,-1.141]
Constant	3.342***	[3.041,3.643]	8.697***	[7.250,10.143]	246.489***	[229.647,263.330]

N = 2594.

^a^ The indexes were mean centered to reduce collinearity in the presence of their interaction.

Abbreviations: CI = Confidence Interval, ref = Reference Category

*p* < .05 *

*p* < .01 **

*p* < .001 ***

Post-hoc, exploratory analyses further revealed interaction effects between cardiovascular risk and infection exposure associated with cognitive function when using infection-burden indices based on only virus exposure alone or parasite exposure alone (Table 5).

**Table 5:** Interaction effects of parasite or virus-specific infection-burden indices and a cardiovascular-risk index and association with SDS performance in U.S. adults aged 20 to 59 years .

	Parasites		Viruses	
	B	95% CI	B	95% CI
Infection Burden Index^a^	.081**	[.028,.134]	.040*	[.010,.071]
Cardiovascular Risk Index^a^	.008	[-.010,.026]	.012	[-.010,.035]
Interaction	.038*	[.008,.068]	.019**	[.006,.033]
Age (years)	.031***	[.029,.034]	.030***	[.027,.034]
Female	-.134***	[-.197,-.071]	-.151***	[-.212,-.089]
Race-ethnicity				
Non-Hispanic White (ref)	---	---	---	---
Non-Hispanic Black	.402***	[.337,.467]	.371***	[.301,.440]
Mexican American	.311***	[.207,.415]	.262***	[.156,.367]
Other	.355***	[.186,.524]	.332***	[.166,.498]
Education (years)	-.121***	[-.136,-.107]	-.121***	[-.136,-.106]
Poverty-to-income Ratio	-.041***	[-.055,-.026]	-.039***	[-.053,-.025]
Constant	3.181***	[2.965,3.396]	3.243***	[3.025,3.462]

N = 5662.

^a^ The indexes were mean centered to reduce collinearity in the presence of their interaction.

Abbreviations: SDS = Symbol Digit Substitution, CI = Confidence Interval, ref = Reference Category

*p* < .05 *

*p* < .01 **

*p* < .001 ***

## Discussion

In this study of 5662 young to middle-aged adults, we found that cardiovascular risk moderated the association between exposure to common infectious diseases determined by IgG antibodies and processing speed as assessed with the Symbol Digit Substitution test. In other words, the association of infection burden with Symbol Digit Substitution test score differed by cardiovascular risk status. In participants at zero risk for a cardiovascular event in the next 10 years, there were no differences in processing speed with increasing exposure to infectious disease, whereas participants with higher risk for a cardiovascular event had worse processing speed with increased exposure to infectious disease. Thus, cardiovascular risk moderated the association of exposure to infectious disease with cognitive function [25, 34, 35] in young to middle-aged adults.

The association we found between the infection burden-cardiovascular risk interaction and cognitive function is broadly consistent with an earlier study that found an association between a viral burden and cognitive deficits in an elderly sample with cardiovascular disease [36]. However, the study did not investigate interactions between viral burden and cardiovascular disease. The interaction we found between risk factors for a cardiovascular event and the infection index is also consistent with the hypothesis that risk factors for cardiovascular disease might disrupt the integrity of the blood-brain barrier and enable increased penetration of infectious agents into the brain, eventually leading to the development of dementia [37].

Due to the cross-sectional design of our study, we are unable to ascertain whether the interaction between cardiovascular risk and infection burden predicting performance on the Symbol Digit Substitution test is associated with subsequent cognitive impairment such as dementia. However, previous findings have found that low cognitive function at age 18 years is associated with an increased risk of subsequent mild cognitive impairment and early dementia [38], indicating the need for additional work investigating associations between cardiovascular risk and infection burden in early and middle adulthood and later cognitive impairment.

In our study, we found that an interaction between risk for a cardiovascular event and an infection index was associated with cognitive deficits on one of three cognitive tasks. Each of the tests of cognitive function we included evaluates different aspects of cognitive functioning. From our findings, it appears that the interaction between infection burden and cardiovascular risk is associated with some but not necessarily all domains of cognitive functioning. In this regard, Symbol Digit Substitution is a more general measure of cognitive functioning in that this measure of processing speed includes some components of the Serial Digit Learning and Reaction Time tasks, each of which assesses more specific components of cognitive functioning than does the Symbol Digit Substitution test. Thus, it has been suggested that measures of processing speed may reflect overall cognitive function [39]. As such, these findings might relate to more global cognitive function, and the Symbol Digit Substitution test might be better suited to capture that effect than does either the Serial Digit Learning or the simple Reaction Time tests. However, the few available tests of cognitive function in the dataset we used preclude identification of a cognitive profile associated with the interaction between infection burden and cardiovascular risk.

Although Tables 3 and 4 suggest the possibility that the interaction between the infection-burden index and cardiovascular risk was present in women but not in men, a post-hoc three-way interaction analysis did not show a statistically significant difference between women and men in this regard. However, evidence for sex differences in many aspects of infectious diseases including prevalence and immune response to pathogens [40], sex differences in susceptibility to deficits in cognitive performance following exposure to certain infectious diseases [41, 42], sex differences in cardiovascular disease [43], sex differences in Alzheimer’s disease-related pathology [44], and sex differences in cognition in healthy adults and in those with dementia [45] warrant future consideration of sex-specific effects when considering associations between cardiovascular risk, exposure to infection, and cognitive function.

We also carried out post-hoc analyses to determine if the interaction between infectious disease pathogen type (that is, parasitic or viral) and cardiovascular risk might be associated with cognitive function similarly. The results of these analyses showed associations between infectious disease burden and cardiovascular risk regardless of pathogen type. Future research may help determine if specific pathogens might have stronger associations with cognitive outcome in the context of cardiovascular risk or if specific combinations of pathogens might be of greater importance clinically. Similarly, future research might help determine if specific levels of cardiovascular disease might be associated with cognitive outcomes as a guide to clinicians.

Although we did not design our study to investigate mechanisms whereby cardiovascular risk moderates associations between infection burden and cognitive function, the interaction we found associated with processing speed is consistent with the hypothesis that risk factors for cardiovascular disease might disrupt the integrity of the blood-brain barrier and enable increased penetration of infectious agents into the brain, ultimately increasing risk for dementia [37]. In this regard, infectious pathogens in animal models can result in amyloid beta production, which might be both protective against and a risk for Alzheimer’s disease [46]. Further, bacteria themselves can disrupt the integrity of the blood-brain barrier [18]. In our study, we found this interaction associated with processing speed in young and middle-aged adults, suggesting that the interaction between risk factors for a cardiovascular event and infectious diseases might begin well before older age.

Increasing evidence suggests that infectious pathogens are associated with Alzheimer’s disease [47] and that neuroinflammation might play an important role in this association [48]. One potential mechanism for the neuropathology associated with Alzheimer’s is cytokine transport across the blood-brain barrier [49]. In fact, blood-brain barrier dysfunction has been associated with various neurodegenerative conditions [50]. There is also evidence implicating neurovascular dysfunction in blood-brain barrier integrity and associated neuroinflammation [51]. Although aging is associated with disruption of the blood-brain barrier [52], which could potentially lead to increased pathogen penetration, the relatively young age range (20 to 59 years) of the participants we included in this study suggests that aging is not a factor in our results. In that the participants in the dataset we used were young to middle-aged adults, a neurodegenerative disease such as Alzheimer’s disease might not directly relate to our findings. However, a recent review presented evidence of blood-brain barrier disruption in diabetes, possibly associated with neurovascular injury, and how this relates to the cognitive difficulties associated with diabetes [53]. Similarly, blood-brain barrier changes associated with diabetes complicate recovery from stroke [54]. Finally, another study found that both dementia and diabetes but not apolipoprotein E genotype or amyloid pathology were associated with blood-brain barrier permeability [20]. Obesity has been associated with changes in the blood-brain barrier, possibly due to altered peptide transport [55].

Several factors require consideration in interpreting the findings of this study. The cross-sectional design does not allow a determination of the time sequence of events, which is a critical component of establishing causation. However, the cross-sectional design does enable an estimate of the strength of the association between the interaction with infection burden and cardiovascular risk and cognitive function and suggests a dose-response relationship, which are other aspects of investigating causality. Furthermore, the cross-sectional nature precluded knowing the time of original infection, whether any subject had experienced a reactivation of an infection acquired earlier, or determination of when cardiovascular risk factors were first present, all of which could affect our results. Because the design was cross-sectional and not randomized, variables for which we were unable to control might have influenced our findings, resulting in the possibility of residual confounding. Additionally, information from only a limited number of pathogens was available to include in our analyses, and we had to limit our analyses to participants that had also completed the cognitive testing. Other infectious pathogens might have interacted with the pathogens we included in unknown ways. The limited cognitive measures included in the NHANES datasets further constrain the interpretation of our findings in that these measures are brief and do not provide a comprehensive neurocognitive assessment. We also found an association with only one of the three cognitive measures we assessed, suggesting that our results are not confirmatory but instead hypothesis generating. Another limitation was the age of participants (20 to 59 years); older adults have a higher prevalence of both cardiovascular and infectious disease and perhaps a longer time for these diseases to have an effect. We conducted multiple comparisons, which can lead to type-1 errors. However, we protected against type-1 errors with the Benjamini-Hochberg test [33] to minimize risk of type-1 errors. Finally, as the NHANES III collected data from 1988 to 1994, the data might not be representative of the current US population.

In conclusion, we found that cardiovascular risk moderates the association between infectious diseases and processing speed. Participants at low risk for a cardiovascular event in the next 10 years had no differences in processing speed with increasing exposure to infectious disease, whereas participants with risk for a cardiovascular event had worse processing speed with increased exposure to infectious disease. Although our analyses cannot directly address it, our findings are broadly consistent with the hypothesis that cardiovascular risk factors might alter the integrity and function of the blood-brain barrier resulting in increased penetration of infectious diseases into the brain with subsequent changes in cognitive function.
